# Dietary Intake of Methionine, Cysteine, and Protein and Urinary Arsenic Excretion in Bangladesh

**DOI:** 10.1289/ehp.11589

**Published:** 2008-08-22

**Authors:** Julia E. Heck, Jeri W. Nieves, Yu Chen, Faruque Parvez, Paul W. Brandt-Rauf, Joseph H. Graziano, Vesna Slavkovich, Geoffrey R. Howe, Habibul Ahsan

**Affiliations:** 1 Department of Epidemiology, Mailman School of Public Health and; 2 Institute for Social and Economic Research and Policy, Columbia University, New York, New York, USA;; 3 Department of Environmental Medicine, New York University School of Medicine, New York, New York, USA;; 4 Department of Environmental Health Sciences, Mailman School of Public Health, Columbia University, New York, New York, USA;; 5 Department of Health Studies and Cancer Research Center, University of Chicago, Chicago, Illinois, USA

**Keywords:** amino acids, arsenic, Bangladesh, cysteine, diet, dietary protein, methionine, nutrition

## Abstract

**Background:**

In Bangladesh, millions of people are exposed to arsenic in drinking water; arsenic is associated with increased risk of cancer. Once ingested, arsenic is metabolized via methylation and excreted in urine. Knowledge about nutritional factors affecting individual variation in methylation is limited.

**Objectives:**

The purpose of this study was to examine associations between intakes of protein, methionine, and cysteine total urinary arsenic in a large population-based sample.

**Methods:**

The study subjects were 10,402 disease-free residents of Araihazar, Bangladesh, who participated in the Health Effects of Arsenic Longitudinal Study (HEALS). Food intakes were assessed using a validated food frequency questionnaire developed for the study population. Nutrient composition was determined by using the U.S. Department of Agriculture National Nutrient Database for Standard Reference. Generalized estimating equations were used to examine association between total urinary arsenic across quintiles of nutrient intakes while controlling for arsenic exposure from drinking water and other predictors of urinary arsenic.

**Results:**

Greater intakes of protein, methionine, and cysteine were associated with 10–15% greater total urinary arsenic excretion, after controlling for total energy intake, body weight, sex, age, tobacco use, and intake of some other nutrients.

**Conclusions:**

Given previously reported risks between lower rates of arsenic excretion and increased rates of cancer, these findings support the role of nutrition in preventing arsenic-related disease.

In Bangladesh, exposure to arsenic through drinking water is an unprecedented health crisis. The vast majority of Bangladeshis obtain their drinking water through hand-pumped tube wells, one-third of which have been found in recent testing to be contaminated with concentrations > 10 μg/L, the maximum safe standard set by the World Health Organization (British Geological Survey 2001). Exposure to arsenic from drinking water has been associated with skin, liver, kidney, and bladder cancers, as well as cardiovascular, gastrointestinal, develop mental, hematologic, and neurologic effects [Chen et al. 2006b; Hafeman et al. 2005; Heck et al. 2008a; International Agency for Research on Cancer (IARC) 2004b]. The result of the ongoing arsenic exposure is expected to double the cancer rates in Bangladesh (Chen and Ahsan 2004).

Inorganic arsenic ingested in drinking water is rapidly and almost totally (80–90%) absorbed by humans and animals (National Research Council 2001). Arsenic is metabolized primarily in the liver through alternating steps of reduction and methylation, beginning with the reduction of arsenate to arsenite. which is then converted to the intermediate products monomethylarsonous acid and dimethylarsinic acid. Once ingested, the human body readily excretes most of the arsenic, primarily in urine. The biochemical pathways involved in arsenic methylation are dependent on availability of *S*-adenosylmethionine (SAM). The methyl group from SAM may be derived from dietary components such as methionine, choline, folate, and other nutrients. In animal bio-assays, dietary methionine deficiency decreased urinary arsenic excretion and increased tissue retention of arsenic (Maiti and Chatterjee 2000; Vahter and Marafante 1987).

Because of the influence of diet on methylation, arsenic toxicity may be greater among those with poorer diets. Descriptive epidemiologic studies of populations with cutaneous or other health effects of arsenic have reported associations with diets low in animal products and vegetables and high in starches, with low protein consumption (Hsueh et al. 1995; Yang and Blackwell 1961; Zaldivar et al. 1978). Deleterious effects are also seen with arsenic among animals fed low-protein and methionine diets (Hoffman et al. 1992; Lammon and Hood 2004; Maiti and Chatterjee 2001; Vahter and Marafante 1987). However, epidemiologic studies of humans have found mixed results (Chen et al. 1988; Chung et al. 2006; Heck et al. 2007; Hopenhayn-Rich et al. 1996a; Mitra et al. 2004; Smith et al. 2000; Steinmaus et al. 2005). Given that malnutrition is common in Bangladesh, with one-third to one-half of Bangladeshis below optimal body weights, additional studies of diet and arsenic exposure are needed (Ahsan et al. 2006a). The purpose of this study was to examine the effect of dietary protein, methionine, and cysteine on urinary excretion of total arsenic. We hypothesized that these dietary factors would improve excretion of arsenic, resulting in increased levels of arsenic in the urine.

## Materials and Methods

### Health effects of arsenic longitudinal study

The Health Effects of Arsenic Longitudinal Study (HEALS) was established in 2000 in the Araihazar area of Bangladesh to prospectively examine the relationship between arsenic intake and the incidence of cancers, reproductive health, and children’s cognitive development (Ahsan et al. 2006a). In the study area within Araihazar, a defined region of 25 km^2^, 5,966 tube wells were tested for the presence of arsenic; the wells showed a range of arsenic exposures from 0.1 to 860 μg/L (Ahsan et al. 2006b). A precohort survey enumerated 65,000 individual users of these wells. Using this roster, a total of 12,050 eligible individuals were approached for recruitment into the cohort study, and 97.5% agreed to participate, yielding a cohort of 11,746 adult men and women. As many rural Bangladeshis are not able to read, verbal consent was obtained prior to participation. All subjects were between 18 and 76 years of age. Study eligibility required that subjects were married and had lived in their *bari* (cluster of homes) for at least 5 years, to ensure stability of residence. Standardized interviews were conducted in Bengali by trained interviewers, and subjects underwent screening by a trained physician to examine overall and arsenic-specific health conditions. The study was approved by the human subjects protection board of Columbia University and by the Ethical Committee of the Bangladesh Medical Research Council.

For the present study, subjects were excluded if they were missing information on dietary intakes. Because the presence of arsenic-related skin lesions may alter urinary arsenic excretion (Del Razo et al. 1997), subjects with preexisting skin lesions were additionally excluded, as were subjects with incomplete skin lesion assessment.

Information was collected on demographic and socioeconomic variables, tube well use, and lifestyle characteristics. Socioeconomic variables included occupation, education, and television and land ownership, which have been used in other studies in Bangladesh to indicate wealth (Argos et al. 2007). Occupations were stratified into higher energy expenditure (daily laborer, farmer, factory worker) and lower energy expenditure (business, unemployed, homemaker, or other).

### Dietary assessment

The study team conducted focus groups to ascertain the range of foods common among diets in this region. Based on the results of these pilot studies, a 39-item food frequency questionnaire (FFQ) was designed that included foods with intakes of at least once per month. The FFQ asked how often, on average, subjects had consumed a particular food item during the previous 12 months, specifying the number of months of the year, the number of days in a week, the number of times a day, and the serving size. Nutrient composition of foods was taken from the U.S. Department of Agriculture (USDA) database for standard reference (USDA 2004). Of all subjects completing the questionnaire, 97% were not missing data on any food item. There were no significant differences between subjects who missed at least one food item and those who did not with regard to age, sex, socio economic status (SES), or body size (data not shown). Dietary protein was measured as grams of protein per kilogram body weight per day, whereas methionine and cysteine were measured as milligrams per kilogram body weight per day; quintiles were created of these dietary exposures for use in regression analyses.

The validity of protein intake in the FFQ was previously examined in a subgroup of 189 subjects who completed food diaries for 2 weeks (Chen et al. 2004); we conducted additional analyses to examine the validity of amino acid intakes. Pearson correlation coefficients, corrected from within-person error, were 0.53 for protein, 0.48 for methionine, and 0.98 for cysteine. As in other countries with few resources available for food storage or distribution, this region of Bangladesh has significant dietary variability across seasons, and it is likely that this seasonal variability may have contributed to poor correlation for some food items.

### Arsenic measurement

Total arsenic concentrations of well water and urinary arsenic were measured. Total arsenic concentration was determined by graphite furnace atomic-absorption spectrometry (GFAA) with a Hitachi Z-8200 system (Hitachi, Tokyo, Japan), which had a detection limit of 5 μg/L (Van Geen et al. 2003). Water samples found to have an arsenic concentration < 5 μg/L were subsequently reanalyzed by inductively coupled plasma-mass spectrometry, which has a detection limit of 0.1 μg/L. For participants who reported drinking water from more than one well, information was collected on the proportion of drinking water from each well. We derived time-weighted arsenic concentration (TWA) as a function of drinking water duration from each well and well arsenic concentration: TWA in micrograms per liter = ∑ *C*_i_*T*_i_/∑*T*_i_, where *C*_i_ and *T*_i_ denote the well arsenic concentration and drinking duration from each well, respectively (Ahsan et al. 2006b).

A spot urine sample was collected from each cohort member at the time of the physician examination. Total urinary arsenic was measured by GFAA, using a PerkinElmer Analyst 600 graphite furnace system (PerkinElmer, Wellesley, MA), as previously described (Nixon et al. 1991). The measurement of chemicals in urine is frequently adjusted for urinary creatinine to account for variance in hydration. Hydration was controlled by expressing urinary arsenic per gram of creatinine, which was analyzed using a colorimetric Sigma Diagnostics Kit (Sigma Diagnostics, St. Louis, MO). Because of the strong relationship of creatinine to meat consumption (Jacobsen et al. 1979), we conducted additional sensitivity analyses without adjustment for creatinine to examine the stability of study findings; individuals whose creatinine concentrations were < 30 or > 300 mg/dL were excluded (30% of subjects) (Barr et al. 2005).

In studies of nutrition and arsenic, care must be taken when adjusting urinary arsenic measures for creatinine concentration. Urinary creatinine varies by season, age, body size, and sex and is related to protein intake, muscle mass, and nutritional status (Barr et al. 2005; Gamble and Liu 2005). Creatinine concentrations in Bangladesh are likely to be lower than those seen in western settings because of higher rates of malnutrition, suggesting that creatinine-adjusted urinary measures will be higher than those seen in other populations (Nermell et al. 2008). Urinary measures of creatinine and arsenic have been correlated in some studies, suggesting that control for creatinine will result in an underestimation of true urinary arsenic concentrations. For these reasons, we provided urinary arsenic concentrations both adjusted and unadjusted for creatinine.

### Statistical analyses

Our dependent variable was total urinary arsenic. Measures of mean values of arsenic exposure, including well water arsenic concentration, time-weighted arsenic exposure, urinary arsenic, and hydration-adjusted urinary arsenic, were compared across subjects by demographic, socioeconomic, and nutrition variables.

Analyses examined the association between dietary exposures with urinary arsenic while controlling for confounding variables. Variables considered as confounding factors in the model had been previously associated with arsenic metabolism and arsenic-related health effects, including arsenic exposure (well water arsenic concentration and time-weighted arsenic), sex, age, tobacco use, and the socioeconomic measures of education level, land ownership, and television ownership (Ahsan et al. 2006b; Argos et al. 2007; Chen et al. 2006a; Heck et al. 2007).

Pyroxidine (vitamin B_6_) and riboflavin (vitamin B_2_) have been identified as necessary for protein metabolism, whereas folate and serine are necessary for amino acid metabolism; these variables were included in analyses as mediators (Bailey and Gregory 1999; Bro-Rasmussen and Horwitt 1967; Huang et al. 1998; Porrini et al. 1989; Shoveller et al. 2005). Other nutrients hypothesized to ameliorate arsenicosis, including folate, calcium, iron, and Vitamin C, were considered potential confounders in analyses (Mitra et al. 2004). Intakes of these nutrients were also assessed using the USDA food composition table (USDA 2004) and the above-mentioned FFQ (Chen et al. 2004).

Confounding was assessed in regression analysis. If variables did not change the beta value of the main effects by 10%, they were not included in the final model.

Because subjects included married couples sharing the same well, we used generalized estimating equation (GEE) analysis to account for the correlation between subjects’ arsenic exposure and other variables.

## Results

Of the 11,746 subjects in the cohort, 352 (3.0%) subjects who were lacking information on dietary intakes were excluded from the current analysis. In the manner suggested by Willett (1990), we excluded 224 (1.9%) additional subjects whose total caloric intake was > 3,500 kcal/day (women) or > 4,000 kcal/ day (men), or < 500 kcal/day (women) or < 800 kcal/day (men). An additional 679 subjects with prevalent skin lesions were excluded, as well as 89 subjects with incomplete skin lesion assessment. The final sample size was 10,402 subjects.

Demographic and socioeconomic characteristics are shown in Table 1. The excluded group of individuals with prevalent skin lesions was disproportionately older and male; thus, well water arsenic concentrations in the remaining study sample were higher among women and among factors associated with female sex, such as employment in a lower energy expenditure occupation, lower body mass index (BMI), non smoking, and not using betel leaf. In addition, those at a lower SES were exposed to higher average levels of well water arsenic. Urinary arsenic, after controlling for creatinine, was associated with nearly all demographic variables. Higher urinary arsenic was associated with female sex, younger age, lower BMI, and lower education, and it was higher among subjects who did not smoke or use betel leaf. Higher urinary arsenic values were seen with higher dietary consumption of protein, methionine, and cysteine (Table 2).

In GEE analyses, subjects at the highest quintiles of protein, methionine, and cysteine intake had greater urinary arsenic, after controlling for sex, age, tobacco use, folate and riboflavin consumption, urinary creatinine, and exposure to arsenic (Table 3). These findings were stable when measuring arsenic exposure both as well water arsenic concentration and as time-weighted arsenic exposure. When urinary creatinine was not included as a covariate in models, a similar effect could be seen. Values for television and land owner ship, betel leaf use, occupation, vitamins C and B_6_, and calcium did not change the main effects beta values by 10% and so were not retained in the regression analysis as confounding factors.

## Conclusions

In this study we found that greater intakes of protein and amino acids resulted in increased urinary excretion of arsenic. Those whose protein consumption exceeded 1.87 g/kg/day had higher urinary arsenic, even after controlling for arsenic intake through drinking water. Those with methionine and cysteine consumption in the highest quintile also experienced this positive effect. To our knowledge, the effect of protein and/or amino acids on total urinary arsenic has not previously been reported in humans. In a study in rabbits injected with 0.4 mg arsenite/kg body weight, those fed diets low in protein, methionine, or choline had a 20% reduction in total urinary arsenic (Vahter and Marafante 1987). In that study, rabbits on the low methionine diet had the greatest reduction in methylating capacity. In the current study, greater methionine and cysteine consumption were associated with comparable increases in total urinary arsenic.

Our results support other research that has found that dietary factors alter urinary arsenic excretion. Nutrient intake is associated with changes in urinary arsenic metabolites, with greater intake of cysteine associated with increased excretion of dimethylarsinic acid (Heck et al. 2007). There is interindividual variation in arsenic excretion, which may be modified by host factors such as age and physical activity level (Vahter et al. 2006). Folate deficiency has been associated with impaired arsenic methylation in participants of our HEALS study, and folic acid supplementation has improved methylation (Gamble et al. 2006). Age influences arsenic metabolism, with urinary arsenic concentrations peaking at younger ages and poorer methylation among older people (Hsueh et al. 1998; Kurttio et al. 1998). Lifestyle differences such as smoking and alcohol use also affect arsenic speciation and total urinary arsenic (Hopenhayn-Rich et al. 1996b; Kurttio et al. 1998).

The higher urinary arsenic found in this study among those with higher protein and amino acid consumption is likely to be explained by the roles of these nutrients in arsenic methylation. Experimental research in animals has found that low protein and amino acid diets increase risks of arsenic-related health effects, and some studies in humans have found worsened arsenic-associated health effects among those consuming lower amounts of meat, eggs, and vegetables (Chen et al. 1988; Hoffman et al. 1992; Lammon and Hood 2004; Maiti and Chatterjee 2001; Mitra et al. 2004; Vahter and Marafante 1987; Yang and Blackwell 1961). However, some studies of protein, amino acids, and arsenic-related skin lesions found little or no effect. Variation in results across studies is likely due to lack of information on individual exposure to both nutrients and arsenic (Hopenhayn-Rich et al. 1996a; Smith et al. 2000); the use of pregnant women as study subjects, who appear to have differing arsenic metabolism than nonpregnant adults (Li et al. 2008); and the use of patients with prevalent skin lesions as study subjects (Chung et al. 2006), as they have altered metabolism and excretion of arsenic.

The population of this study was exposed to a wide range of arsenic concentrations in well water. Subjects experienced differential exposure to well water concentrations of arsenic by SES. Previous research found access to and use of a sanitary water supply (tube wells) in Bangladesh to be strongly related to SES measures such as income and education (Taha et al. 2000; Vahter et al. 2006; Yusuf and Zakir Hussain 1990). Greater arsenic concentrations in wells used by those at lower income may occur because of differing well depths among study participants; wealthier subjects are more likely to have deeper wells, which tend to cost more and have lower arsenic concentrations. Arsenicosis is more prevalent among poorer Bangladeshis, which has previously been attributed to differences in nutrition and access to treatment (Hadi and Parveen 2004; Sikder et al. 2005).

The most common dietary sources in Bangladesh for protein, methionine, and cysteine come from rice and fish (Ahmad et al. 1986; Roos et al. 2003). In Bangladesh, as in other countries in Asia and elsewhere, intake of protein and amino acids depends on demographic characteristics such as age, sex, and SES (Heck et al. 2008b). Studies of food intake have shown that Bangladeshi men consume greater amounts of meats, eggs, pulses, milk, vegetables, and fruits than Bangladeshi women (Sudo et al. 2004). However, there are reports of both male and female Bangladeshis suffering from chronic energy deficiency (Abdullah et al. 1995; Choudhury et al. 2000; Mitra 1997). Age is also related to protein intake, with adults typically consuming less protein as they age (Smit et al. 1999). If protein sources are scarce, protein may be given to honored family members or those undertaking the greatest physical labor. Energy intake is also strongly associated with SES measures such as income or education (Ahmad et al. 1986). These underlying differences must be taken into account when assessing disease risk.

There was considerable undernutrition among subjects in this study, as evidenced by the low BMI scores. Although low BMI independently predicts arsenic-related health effects, it is unlikely to explain the findings here, as protein, methionine, and cysteine consumption were expressed in units per body weight (Guha Mazumder et al. 1998; Milton et al. 2004). The frequent co-occurrence of dietary protein with other important nutrients such as iron, calcium, iodine, and vitamins A, B_1_, niacin, B_12_, and folate suggests that those subjects at the highest quintile of protein consumption may have had better diets overall, contributing to better overall health. Nonetheless, our analysis controlled for folate, iron, serine, and riboflavin, and we found little effect from calcium, iron, vitamin C, and vitamin B_6_, and as such, these factors are unlikely to have explained the results.

Trace amounts of arsenic are found in tobacco. Studies of cigarettes manufactured in the United States estimate arsenic concentrations per cigarette at 10.7 ng (range, 1.6–24.9 ng), which are attributed to pesticide residues (IARC 2004a). Little is known about arsenic concentrations of tobacco grown in Bangladesh, although levels may vary depending on pesticide use and whether arsenic-contaminated water is used in crop irrigation. An individual’s exposure may also depend on whether the tobacco is smoked or chewed. Bangladeshis may be similarly exposed to trace amounts of arsenic from food intake because of contaminated water used in crop irrigation or in cooking. Nonetheless, it is expected that the majority of arsenic intake among HEALS subjects comes from drinking-water exposure, with amounts from other sources expected to be negligible (Ahsan et al. 2006a). In this study, similar patterns of urinary excretion were seen between smokers and nonsmokers (data not shown).

Accurate assessment of dietary intake is vital in nutritional studies. All FFQs have strengths and limitations and are subject to error. The validation study of the FFQ used in this study found some nutrient estimates to be overestimated compared with 7-day food diaries. Despite the fact that many nutrients had reasonable correlations between FFQ and food diary, we cannot exclude the possibility that subjects overestimated their consumption, especially for foods with greater social desirability such as tea and fruit (Chen et al. 2004).

In the present study, we did not evaluate the influence of arsenic metabolism capacity on urinary arsenic excretion. Arsenic metabolism capacity may be in the causal pathway of nutritional status urinary excretion ability. Future studies on this topic are needed. In separate analyses, we examined the nutritional influence on arsenic metabolism capacity indicated using urinary arsenic metabolite profile (Heck et al. 2007).

In this study we found significant differences in total urinary arsenic among those consuming varying amounts of dietary protein, methionine, and cysteine, even after controlling for arsenic exposure. Given the potential protective effect of these nutrients, the extent of undernutrition in Bangladesh is of even greater concern. Programs designed to alleviate the suffering of arsenicosis patients in Bangladesh may consider dietary supplementation.

## Figures and Tables

**Table 1:** Population characteristics and mean ± SD values of water arsenic, time-weighted well water arsenic, urinary arsenic (U-As), and U-As per gram creatinine, by sociodemographic variables.

	No. (%)	Protein (g/kg bw/day)	Methionine (mg/kg bw/day)	Cystine (mg/kg bw/day)	Well water As concentration (μg/L)	Time-weighted well water As (duration × μg/L)	U-As (μg/L)	U-As (μg)/ g creatinine (μg/L)
Sex								
Female	6,264 (60.2)	1.54 ± 0.44	34.4 ± 10.5	18.3 ± 5.2	98.4 ± 111.1	97.4 ± 107.3	132.3 ± 152.2	297.6 ± 289.4
Male	4,138 (39.8)	1.57 ± 0.43	35.0 ± 10.3	19.1 ± 5.2	93.6 ± 106.0	91.8 ± 101.7	131.7 ± 137.8	229.8 ± 249.0
* p*-Value		0.0008	0.003	< 0.0001	0.03	0.009	< 0.0001	< 0.0001
Age (years)								
18–30	2,756 (26.5)	1.59 ± 0.42	35.8 ± 10.2	19.0 ± 5.0	98.6 ± 111.1	97.0 ± 107.6	142.0 ± 163.3	301.1 ± 304.7
30–39	3,736 (35.9)	1.55 ± 0.43	34.7 ± 10.3	18.6 ± 5.2	95.1 ± 107.9	95.0 ± 104.3	129.1 ± 141.7	265.3 ± 276.3
40–49	2,587 (24.9)	1.53 ± 0.44	33.9 ± 10.6	18.4 ± 5.4	96.9 ± 109.7	94.5 ± 104.0	127.3 ± 141.0	261.4 ± 264.6
≥ 50	1,323 (12.7)	1.52 ± 0.45	33.6 ± 10.7	18.4 ± 5.5	95.3 ± 107.4	93.1 ± 104.7	128.8 ± 133.2	239.3 ± 223.7
* p*-Value		< 0.0001	< 0.0001	< 0.0001	0.6	0.7	0.0008	< 0.0001
BMI								
< 18.5	3,957 (39.0)	1.68 ± 0.47	37.4 ± 11.4	20.1 ± 5.7	99.7 ± 111.1	98.2 ± 106.7	139.9 ± 156.2	301.3 ± 291.2
18.5–20.5	2,718 (26.8)	1.56 ± 0.40	34.9 ± 9.6	18.7 ± 4.8	96.9 ± 108.9	95.3 ± 103.8	131.0 ± 141.7	262.7 ± 247.4
20.5–22.5	1,653 (16.3)	1.46 ± 0.37	32.8 ± 9.0	17.6 ± 4.5	99.9 ± 114.5	98.0 ± 109.7	126.7 ± 143.2	266.7 ± 261.6
22.5–25	1,085 (10.7)	1.38 ± 0.34	31.2 ± 8.4	16.7 ± 4.1	87.3 ± 100.4	86.7 ± 100.0	120.7 ± 129.4	234.1 ± 330.4
≥ 25.0	723 (7.1)	1.25 ± 0.33	28.2 ± 8.1	15.4 ± 4.2	83.3 ± 101.5	84.3 ± 101.6	125.3 ± 144.1	197.4 ± 206.2
* p*-Value		< 0.0001	< 0.0001	< 0.0001	< 0.0001	0.001	0.0003	< 0.0001
Education (years)								
No formal	4,608 (44.3)	1.54 ± 0.44	34.3 ± 10.6	18.4 ± 5.2	95.6 ± 106.8	93.9 ± 102.2	135.9 ± 146.9	288.4 ± 266.4
1–5	3,068 (29.5)	1.57 ± 0.43	34.9 ± 10.3	18.8 ± 5.2	99.8 ± 113.4	97.1 ± 107.8	137.3 ± 153.5	276.7 ± 262.0
6–10	2,306 (22.2)	1.56 ± 0.43	35.1 ± 10.4	18.8 ± 5.2	96.0 ± 109.7	96.7 ± 108.8	122.1 ± 147.4	243.4 ± 320.4
≥ 10	415 (4.0)	1.53 ± 0.42	34.6 ± 10.3	18.9 ± 5.2	85.1 ± 97.8	86.8 ± 97.2	107.5 ± 118.3	183.7 ± 165.4
* p*-Value		0.02	0.02	0.0009	0.05	0.2	< 0.0001	< 0.0001
Occupation								
Higher energy	7,784 (74.8)	1.56 ± 0.44	34.8 ± 10.4	18.5 ± 5.2	88.7 ± 101.2	87.4 ± 98.5	124.5 ± 130.3	214.9 ± 252.1
Lower energy	2,617 (25.2)	1.53 ± 0.43	34.2 ± 10.4	18.9 ± 5.4	99.1 ± 112.1	97.7 ± 107.1	134.6 ± 153.2	289.5 ± 281.7
* p*-Value		0.005	0.01	0.008	< 0.0001	0.003	0.003	< 0.0001
Land ownership								
No	5,201 (50.0)	1.54 ± 0.44	34.2 ± 10.6	18.5 ± 5.3	99.6 ± 111.8	97.4 ± 107.5	135.0 ± 154.0	278.1 ± 264.9
Yes	5,198 (50.0)	1.56 ± 0.43	35.1 ± 10.2	18.8 ± 5.2	93.4 ± 106.3	93.0 ± 102.7	129.2 ± 138.9	263.0 ± 286.3
* p*-Value		0.002	< 0.0001	0.001	0.004	0.03	0.05	0.007
TV ownership								
No	6,766 (65.0)	1.55 ± 0.43	34.5 ± 10.4	18.6 ± 5.2	100.7 ± 112.9	98.8 ± 108.0	137.3 ± 150.6	287.9 ± 271.6
Yes	3,636 (35.0)	1.55 ± 0.44	34.5 ± 10.5	18.7 ± 5.3	88.8 ± 101.3	88.4 ± 99.2	122.9 ± 138.6	239.1 ± 280.9
* p*-Value		0.8	0.1	0.1	< 0.0001	< 0.0001	< 0.0001	< 0.0001
Smoking								
No	7,616 (73.2)	1.54 ± 0.43	34.4 ± 10.4	18.3 ± 5.1	98.7 ± 111.2	97.6 ± 107.4	133.2 ± 150.7	282.6 ± 294.2
Yes	2,785 (26.8)	1.59 ± 0.43	35.4 ± 10.4	19.4 ± 5.3	90.5 ± 103.0	88.3 ± 98.3	128.8 ± 134.9	237.6 ± 215.5
* p*-Value		< 0.0001	< 0.0001	< 0.0001	0.0004	< 0.0001	0.2	< 0.0001
Betel leaf use								
No	6,785 (65.2)	1.56 ± 0.42	34.8 ± 10.2	18.7 ± 5.1	98.2 ± 110.4	96.8 ± 107.1	134.9 ± 149.9	275.0 ± 290.0
Yes	3,616 (34.8)	1.54 ± 0.45	34.3 ± 10.9	18.5 ± 5.5	93.4 ± 106.7	92.0 ± 101.3	126.8 ± 140.1	262.5 ± 247.3
* p*-Value		0.02	0.03	0.1	0.02	0.03	0.007	0.02

Abbreviations: bw, body weight; U-As, urinary arsenic. *p*-Values were computed with analysis of variance.

**Table 2:** Mean values of water arsenic, time-weighted arsenic, urinary arsenic (U-As), and U-As per gram creatinine by quintiles of protein, methionine, and cysteine.

	Well water As concentration (μg/L)		Time-weighted well water As (duration × μg/L)		U-As (μg/L)		U-As (μg)/ g creatinine (μg/L)	
	Mean ± SD	*p*-Trend	Mean ± SD	*p*-Trend	Mean ± SD	*p*-Trend	Mean ± SD	*p*-Trend
Protein (g/kg/day)		0.9		0.8		0.009		0.003
< 1.20	98.7 ± 112.1		97.4 ± 108.9		130.0 ± 134.6		260.2 ± 257.9	
1.20–1.41	93.4 ± 106.6		91.7 ± 103.0		127.5 ± 140.8		264.1 ± 261.4	
1.41–1.61	95.2 ± 108.8		94.3 ± 106.1		128.8 ± 150.2		267.9 ± 272.7	
1.61–1.87	97.8 ± 108.1		95.9 ± 102.7		134.0 ± 143.9		277.3 ± 316.0	
> 1.87	96.6 ± 110.9		96.0 ± 105.8		140.4 ± 162.0		283.1 ± 267.8	
Methionine (mg/kg/day)		0.05		0.08		0.06		0.02
< 26.5	100.0 ± 113.0		99.0 ± 110.1		130.7 ± 137.4		260.1 ± 254.3	
26.5–31.2	97.8 ± 112.6		94.9 ± 106.8		128.3 ± 136.6		270.0 ± 271.4	
31.2–35.7	94.6 ± 107.5		93.9 ± 105.6		128.8 ± 152.0		261.7 ± 246.0	
35.7–41.9	96.1 ± 106.8		95.0 ± 103.2		136.6 ± 153.5		282.0 ± 328.7	
> 41.9	93.3 ± 106.3		92.4 ± 100.6		136.3 ± 152.9		278.8 ± 271.9	
Cysteine (mg/kg/day)		0.7		0.6		0.006		0.03
< 14.4	97.4 ± 112.0		95.9 ± 108.6		129.0 ± 134.5		265.4 ± 262.4	
14.4–16.9	95.0 ± 107.9		93.9 ± 104.9		129.6 ± 141.0		266.0 ± 255.1	
16.9–19.3	94.2 ± 107.9		92.6 ± 104.4		127.2 ± 148.4		264.5 ± 276.0	
19.3–22.4	97.1 ± 106.3		96.2 ± 102.5		133.8 ± 146.1		276.6 ± 315.5	
> 22.4	98.1 ± 112.2		96.6 ± 106.3		141.2 ± 161.7		280.9 ± 266.6	

**Table 3:** Association between dietary exposures and urinary arsenic values (estimates from GEE) by multivariate analysis.

	Models with As exposure measured as index well water concentration						Models with As exposure measured as TWA					
	Creatinine adjusted (*n* = 10,402)			Unadjusted^a^ (*n* = 6,758)			Creatinine adjusted (*n* = 10,402)			Unadjusted^a^ (*n* = 6,758)		
	Least squares adjusted mean	β	*p* -Value	Least squares adjusted mean	β	*p* -Value	Least squares adjusted mean	β	*p* -Value	Least squares adjusted mean	β	*p* -Value
Quintiles of protein consumption^b^ (g/kg/day)												
< 1.20	117.5		Referent	148.2		Referent	115.6		Referent	156.6		Referent
1.20–1.41	130.1	12.7	< 0.0001	163.4	7.1	0.1	127.7	12.1	0.0001	162.3	5.7	0.2
1.41–1.61	132.4	14.9	< 0.0001	166.9	4.7	0.4	130.7	15.1	< 0.0001	160.9	4.3	0.4
1.61–1.87	137.3	19.8	< 0.0001	170.8	7.9	0.1	136.1	20.5	< 0.0001	164.7	8.1	0.1
> 1.87	145.2	27.7	< 0.0001	179.2	13.8	0.03	144.5	28.9	< 0.0001	171.9	15.3	0.02
Quintiles of methionine consumption^c^ (mg/kg/day)												
< 26.5	116.1		Referent	154.9		Referent	114.9		Referent	154.3		Referent
26.5–31.2	125.9	9.9	0.001	159.9	5.0	0.3	124.7	9.7	0.002	158.1	3.8	0.4
31.2–35.7	131.0	15.0	< 0.0001	163.7	8.8	0.09	129.0	14.1	0.0001	161.9	7.6	0.2
35.7–41.9	141.1	25.0	< 0.0001	171.2	16.3	0.004	139.4	24.5	< 0.0001	168.7	14.4	0.01
> 41.9	148.1	32.0	< 0.0001	175.8	20.9	0.002	146.7	31.9	< 0.0001	173.7	19.5	0.003
Quintiles of cysteine consumption^d^ (mg/kg/day)												
< 14.4	116.8		Referent	159.0		Referent	115.1		Referent	157.1		Referent
14.4–16.9	126.9	10.1	0.0009	162.2	3.1	0.5	124.8	9.7	0.002	159.5	2.4	0.6
16.9–19.3	131.8	15.0	< 0.0001	162.8	3.8	0.4	130.1	15.1	< 0.0001	160.4	3.4	0.5
19.3–22.4	138.6	21.8	< 0.0001	167.9	8.8	0.1	137.1	22.0	< 0.0001	166.0	8.9	0.1
> 22.4	148.0	31.2	< 0.0001	173.1	14.1	0.03	147.6	32.5	< 0.0001	173.4	16.3	0.01

a Subjects included had urinary creatinine concentrations between 30 and 300 mg/dL.

b Controlling for arsenic exposure, sex, age, tobacco use, total energy (kcal), education level, land ownership, television ownership, and folate and riboflavin consumption.

c Controlling for arsenic exposure, sex, age, tobacco use, total energy (kcal), education level, land ownership, television ownership, and folate and serine consumption.

d Controlling for arsenic exposure, sex, age, tobacco use, total energy (kcal), education level, land ownership, television ownership, and iron, folate and serine consumption.
